# Dr. Sylvanus E. Parker

**Published:** 1904-05

**Authors:** 


Dr. Sylvanus E. Parker, of Naples, N. Y., died at his home, April 4, 1904, aged 53 years. He graduated at the University of Buffalo in 1877.

